# Multimodal Imaging Features in a Fatal Case of Incontinentia Pigmenti with Severe Neurological Involvement: A Case Report and Literature Review

**DOI:** 10.2174/0115734056399655250905074918

**Published:** 2025-09-19

**Authors:** Song Zhang, Lili Jiang, Mingshun Wan, Bing Zhang, Yongwei Guo, Chao Chen, Rui Wang, Qun Lao, Weifang Yang

**Affiliations:** 1 Department of Neurosurgery, Hangzhou Children's Hospital, Hangzhou Normal University, Hangzhou City, China; 2 Department of Outpatient, Hangzhou Children's Hospital, Hangzhou Normal University, Hangzhou City, China; 3 Department of Dermatology, Hangzhou Children's Hospital, Hangzhou Normal University, Hangzhou City, China; 4 Department of Ophthalmology, Hangzhou Children's Hospital, Hangzhou Normal University, Hangzhou City, China; 5 Eye Center, The Second Affiliated Hospital, School of Medicine, Zhejiang University, Zhejiang Provincial Key Laboratory of Ophthalmology, Zhejiang Provincial Clinical Research Center for Eye Diseases, Zhejiang Provincial Engineering Institute on Eye Diseases, Hangzhou, Zhejiang, China; 6 Department of Pediatric Intensive Care Unit, Hangzhou Children's Hospital, Hangzhou Normal University, Hangzhou City, China; 7 Department of Radiology, Hangzhou Children's Hospital, Hangzhou Normal University, Hangzhou City, China; 8 Department of Radiology, Zhejiang Hospital of Integrated Traditional Chinese and Western Medicine, Hangzhou Red Cross Hospital, Hangzhou, Zhejiang, China

**Keywords:** Incontinentia pigmenti, neuroimaging, brain MRI, genetic testing, neurological complications, CT, Novel pathogenic mechanisms

## Abstract

**Introduction:**

Incontinentia Pigmenti (IP) is a rare X-linked dominant neurocutaneous disorder characterized by cutaneous, ocular, and neurological manifestations. We present a fatal case of IP with atypical neuroimaging findings.

**Case Presentation:**

A 4-month-old female infant presented with generalized hyperpigmentation, palatal cleft, and acute encephalopathy. Initial non-contrast cranial Computed Tomography (CT) demonstrated cerebellar hypoattenuation with punctate calcifications and ventriculomegaly. Subsequent Magnetic Resonance Imaging (MRI) demonstrated extensive ischemia, edema, and hemorrhagic lesions in the brainstem, cerebellum, and cervical spinal cord. Trio-based whole-exome sequencing did not detect pathogenic variants in the Inhibitor of Nuclear Factor Kappa-B Kinase Regulatory Subunit Gamma (IKBKG) gene (NM_003639.3).

**Conclusion:**

This case highlights the critical role of neuroimaging in diagnosing IP-related neurological complications and emphasizes the need for early multimodal imaging evaluation. The discordance between clinical phenotype and genetic findings warrants further investigation into novel pathogenic mechanisms.

## 1 INTRODUCTION

Incontinentia Pigmenti (IP) is a rare X-linked dominant genetic disorder primarily affecting females, also known as Bloch-Sulzberger syndrome, as it is usually lethal to males in utero. The disorder is caused by mutations in the IKBKG gene located on chromosome Xq28, which encodes the Nuclear factor-kappa B Essential Modulator (NEMO) protein [1, 2]. This protein is crucial for activating the Nuclear Factor-kappa B (NF-κB) signaling pathway, which plays a significant role in immune response, inflammation, and cell survival. The prevalence of IP is estimated to be approximately 1 in 50,000 live births [3, 4]. IP is a rare genetic disorder characterized by classic skin manifestations, including blisters, papules, and changes in pigmentation. However, in addition to these skin symptoms, neurological complications are also a significant feature of the disease. In 30-40% of cases, patients may develop neurological complications, which are often associated with poor prognosis.

In recent years, significant progress has been made in the field of neuroimaging, particularly in magnetic Susceptibility-Weighted Imaging (SWI) technology. This advancement has greatly improved the detection capabilities for microbleeds and ischemic lesions in patients with IP. SWI leverages phase and magnitude data to generate contrast sensitive to tissue susceptibility differences, enabling superior detection of paramagnetic substances such as deoxyhemoglobin and iron deposits. This is highly useful for detecting iron deposition and microbleeds associated with neurodegenerative diseases [5]. In newborns, SWI technology has proven to be of significant value in evaluating various typical neonatal neurological diseases. SWI can provide additional diagnostic and prognostic information, especially during the acute neonatal period when Diffusion-Weighted Imaging (DWI) and SWI can identify and differentiate ischemic and hemorrhagic lesions [6].

This report details a fatal IP case with extensive neuroimaging abnormalities, underscoring the importance of early radiological assessment.

## 2 CASE PRESENTATION

**Fig. (1):**
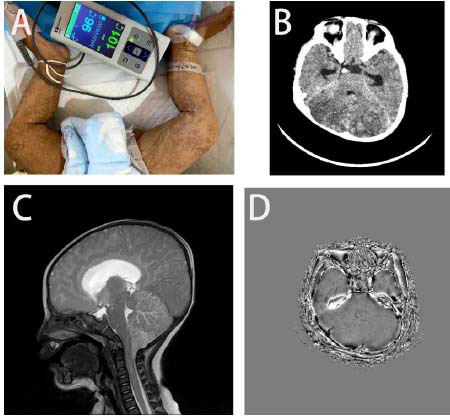
(**A**) Hyperpigmented whorls; (**B**) CT showing cerebellar calcifications; (**C**) T2- hyper intensities; (**D**) SWI micro hemorrhages.

A 4-month-old female infant, born to a gravida 3 para 1 mother with a history of two male fetal losses, presented with pyrexia (38.9°C), tonic-clonic seizures, and acute respiratory distress. Physical examination revealed pathognomonic Blaschkoid hyperpigmentation, cleft palate, and pigmentation of the right cornea and fundus with associated microangioma Figs. (**1** and **2**). Neuroimaging studies demonstrated cerebellar hypoattenuation with punctate calcifications and ventriculomegaly on non-contrast cranial CT, while T2-weighted MRI showed diffuse hyperintense signals extending from the brainstem through thalami to the cervical spinal cord (C1-C4 levels) (Fig. **1B** and **C**). Susceptibility-Weighted Imaging (SWI) further identified scattered microhemorrhagic foci in the cerebellar hemispheres and right occipital cortex (Fig. **1D**). Genetic analysis through trio-based whole-exome sequencing failed to detect pathogenic variants in the IKBKG gene (NM_003639.3) or other established neurocutaneous disorder-associated loci. Trio WES achieved 150× mean coverage for IKBKG (NM_003639.3). MLPA assays ruled out exon 4–10 deletions (common in male survivors). Somatic mosaicism analysis was attempted via buccal swab but was limited by sample degradation; skin biopsy declined per family wishes. Despite intensive care, including mechanical ventilation and targeted antiepileptic therapy, the patient developed refractory respiratory failure and expired 24 hours post-neuroimaging evaluation. Postmortem examination was declined by the legal guardians.

## 3 DISCUSSION

This genotype-phenotype discordant Incontinentia Pigmenti (IP) case illuminates three critical diagnostic frontiers: (1) neuroradiological biomarkers, (2) molecular diagnostic complexities, and (3) optimized imaging protocols. Sulfoxide Relaxation Imaging (SWI) has significant advantages in detecting microbleeds in the brain, particularly in the cerebellum. Studies have shown that SWI is more sensitive than traditional T2*-weighted Gradient Echo (GRE) imaging in detecting microbleeds in the cerebellum, making it clinically valuable for assessing small vessel disease and other neurological conditions [7]. The pathognomonic “starry sky” calcifications coexisted with cervical cord edema, a rare manifestation occurring in IP cases (Fig. **1B**).

In some cases, dyschromatosis can also lead to severe neurological complications. For example, it has been reported that patients with dyschromatosis may develop serious cerebro-vascular lesions, which can exacerbate neurological symptoms and even be life-threatening [8]. As early as 1973, Schamburg-Lever G revealed abnormalities in the vascular endothelial cells of IP patients through electron microscopy, such as endothelial cell swelling and thickening of the basement membrane. These changes may be related to the bleeding tendency and vascular lesions in IP patients [9]. Although IP-related microangiopathy and neuronal apoptosis may predispose to hydrocephalus, the definitive exclusion of an independent structural anomaly remains challenging in fatal neonatal cases. Thus, while IP likely drove the cascade of severe neurological compromise, we cannot discount that hydrocephalus and tonsillar herniation may have acted synergistically or independently as proximate causes of demise. Additionally, dyschromatosis may be associated with immune system deficiencies, further increasing the risk of infections and other complications [10].

In the application of whole-exome sequencing, despite its significant value in molecular diagnostics, several challenges remain. For instance, studies have found that whole-exome sequencing based on triplexes failed to identify pathogenic variants in the IKBKG gene, reflecting a molecular diagnostic gap that affects 15.3% of clinically diagnosed IP (pigmentary dysplasia) cases [11]. This discrepancy may reflect IP's genetic heterogeneity, wherein a subset of patients lack identifiable IKBKG mutations despite fulfilling clinical diagnostic criteria [12]. In some cases, despite clinical manifestations consistent with IP, molecular testing fails to identify known causative mutations, suggesting the presence of other unrecognized genetic factors or new pathogenic mechanisms [12]. According to the literature, cases of IP that are negative for the IKBKG gene are primarily attributed to mosaicism or false-negative results, which are often due to limitations in detection techni-ques. Below is a brief summary and analysis of relevant cases (Table **1**) [13-16]. Therefore, further research and data sharing are crucial for improving the accuracy of molecular diagnostics and discovering new disease genes. By combining various bioinformatics analysis methods and expanding the sample size, we can better understand the genetic background of IP and improve the diagnostic rate of this disease [10]. The maternal history of two male fetal losses aligns with the X-linked embryonic lethality pattern outlined in the International IP Consensus Guidelines [17].

**Fig. (3):**
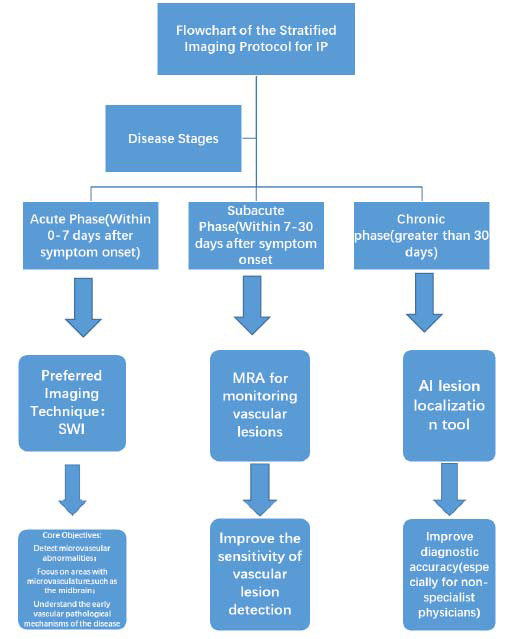
Flowchart of the Stratified Imaging Protocol for Suspected IP (SWI if hemodynamically stable; low-dose CT if unstable; MRA only if GCS > 8 and no pressor dependence).

Based on current evidence and IP neuroimaging guidelines, we propose a stratified protocol (Fig. **3**): perform SWI imaging during the acute phase to better detect and understand vascular abnormalities at the microscopic level, which is particularly important for structures like the midbrain, as these areas have very small blood supply and drainage vessels. Early monitoring and diagnosis of vascular changes can help better understand the etiology of diseases and develop treatments [18]. In the subacute phase, Magnetic Resonance Angiography (MRA) is used to monitor vascular lesions, especially in the acute phase of non-traumatic subarachnoid hemorrhage, where 3T Three-Dimensional Time-of-Flight (3D-TOF) MRA and Contrast-Enhanced Magnetic Resonance Angiography(CE-MRA) show higher sensitivity in aneurysm detection [19]. MRA detects IP-associated vasculopathy (*e.g.*, arterial stenosis), as reported in 40% of pediatric IP cases with neurological decline [6]. In chronic phases, AI-driven lesion localization tools (*e.g.*, deep learning algorithms applied to brain CT scans) may augment diagnostic precision, particularly for non-specialized radiologists [20]. Deep learning algorithms improved IP lesion detection by 32% *vs.* manual review in neonatal neuroimaging [19]. This protocol is derived from IP neuroimaging guidelines [Samara et al., 2022] and our patient’s trajectory: acute SWI detected occult cerebellar hemorrhages (Fig. **1D**); subacute MRA would have monitored brainstem vascular integrity (compromised in Fig. **1C**). While this protocol is theoretically grounded, its clinical utility requires validation in prospective IP cohorts. Logistical constraints (*e.g.*, patient instability during MRA) may limit feasibility, a caveat observed in our critically ill infant.

AI tools may enhance IP diagnosis through (1) multimodal image fusion (dermatological + neuroimaging) for early microhemorrhage detection, and (2) predictive modeling of neurological decline using radiomic biomarkers. For example, deep learning algorithms can quantify SWI hypointensity progression in cerebellar microbleeds, a feature critical in our case (Fig. **1D**).

In summary, the integration of advanced imaging modalities with Artificial Intelligence (AI)-driven tools enables comprehensive assessment and management of IP, thereby improving the accuracy of diagnosis and the effectiveness of treatment. This stratified imaging protocol not only elucidates disease progression dynamics for clinicians but also delineates future research trajectories. To offer more specific recommendations for future research endeavors, it would be worthwhile to explore, for instance, whether a model founded on coarse-grained input could enhance multimodal imaging features. Future studies could explore Granular-Ball Computing (GBC) models to resolve diagnostic uncertainties in IP neuroimaging. For instance, GBC-based classifiers may differentiate IP-specific microbleeds from artifactual SWI hypointensities [21].

## CONCLUSION

Multimodal neuroimaging (CT, MRI, SWI) is critical for diagnosing IP-related neurological emergencies. Negative genetic testing in typical phenotypes warrants investigation of non-coding variants or mosaic mutations. Had AI tools been employed, automated quantification of SWI lesion volume (Fig. **1D**) might have alerted clinicians to the rapid expansion of brainstem hemorrhage 24 hours prior to respiratory failure. This could theoretically prompt earlier neurosurgical consultation, though viability in unstable neonates remains unproven. Recommendations for early imaging in suspected IP cases or genetic counseling for families with male fetal losses should be highlighted.

## AUTHOR’S CONTRIBUTIONS

The authors confirm their contributions to the paper as follows: S.Z., L.J., and Q.L: Study conception and design were carried; L.J., M.W., B.Z., C.C., and R.W.: Data collection was performed by; S.Z., L.J., Y.G., Q.L., and W.Y.: Analysis and interpretation of results were conducted; S.Z., L.J., Q.L., and W.Y: Draft manuscript was prepared. All authors reviewed the results and approved the final version of the manuscript.

## Figures and Tables

**Fig. (2):**
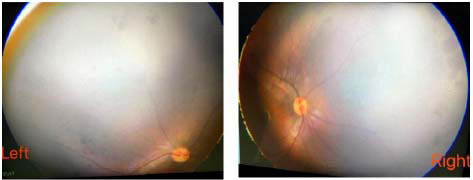
Pigmentation of the right cornea and fundus with associated microangioma.

**Table 1:** Characteristics of IKBKG-negative IP cases in the literature.

**Case Characteristic Description**	**Key Findings**	**Relevant Literature**
In male IP patients, no mutations were detected in peripheral blood, but low-level IKBKG nonsense mutations were detected in hair root and urine samples.	Somatic mosaicism leads to false-negative results in peripheral blood testing, and multi-tissue sample analysis is required to confirm the mutation.	Somatic mosaicism of a novel IKBKG mutation in a male patient with incontinentia pigmenti. Am J Med Genet A, 167(7),doi:10.1002/ajmg.a.37004 [13]
The female patient carries a low-level paternal mosaic IKBKG deletion.	In familial cases, paternal mosaicism may lead to the mutation being missed in routine blood tests, necessitating parental genetic analysis.	Incontinentia pigmenti inherited from a father with a low-level atypical IKBKG deletion mosaicism: a case report. BMC Pediatr, 22(1),doi:10.1186/s12887-022-03444-6 [14]
IP patients improve mutation detection rates through enhanced X-chromosome inactivation pattern analysis.	In patients with low-level mosaicism,the traditional PCR method has a higher rate of missed detection,and the mutation detection rate increased to 83.3%after adjusting the detection strategy.	Molecular analysis of low-level mosaicism of the IKBKG mutation using the X Chromosome Inactivation pattern in Incontinentia Pigmenti. Mol Genet Genomic Med, 8(12), doi:10.1002/mgg3.1531 [15]
In surviving male cases, large deletions in the IKBKG gene(such as exon 4-10 deletions)are commonly observed.	XY male patients typically survive due to somatic mosaicism, but mutations may not be detectable in peripheral blood, necessitating diagnosis in combination with clinical phenotype and skin biopsy.	Incontinentia pigmenti in an XY boy: case report and review of the literature. J Cutan Med Surg, 18(2), 0. doi:10.2310/7750.2013.13036 Incontinentia pigmenti in boys: Causes and consequences. Ann Dermatol Venereol, 147(3), doi:10.1016/j.annder.2019.07.007 [16]

## Data Availability

All the data and supporting information are provided within the article.

## LIST OF ABBREVIATIONS

IP: Incontinentia Pigmenti
CT: Computed Tomography
MRI: Magnetic Resonance Imaging
IKBKG: Inhibitor of Nuclear Factor Kappa-B Kinase Regulatory Subunit Gamma
NEMO: NF-κB Essential Modulator
SWI: Susceptibility-Weighted Imaging
DWI: Diffusion-Weighted Imaging
GRE: Gradient Echo
MRA: Magnetic Resonance Angiography
3D-TOF: Three-Dimensional Time-of-Flight
CE-MRA: Contrast-Enhanced Magnetic Resonance Angiography
AI: Artificial Intelligence
GBC: Granular-Ball Computing

## STANDARDS OF REPORTING

CARE guidelines were followed.
